# Supplementary material for: Glycoalkaloid Composition and Flavonoid Content as Driving Forces of Phytotoxicity in Diploid Potato
Source: Int J Mol Sci. 2023 Jan 14;24(2):1657. doi: 10.3390/ijms24021657 (PMC9863746; doi:10.3390/ijms24021657)
Supplement: Supplementary file 1 [file ijms-24-01657-s001.zip › Supplementary Figure S1.pdf]

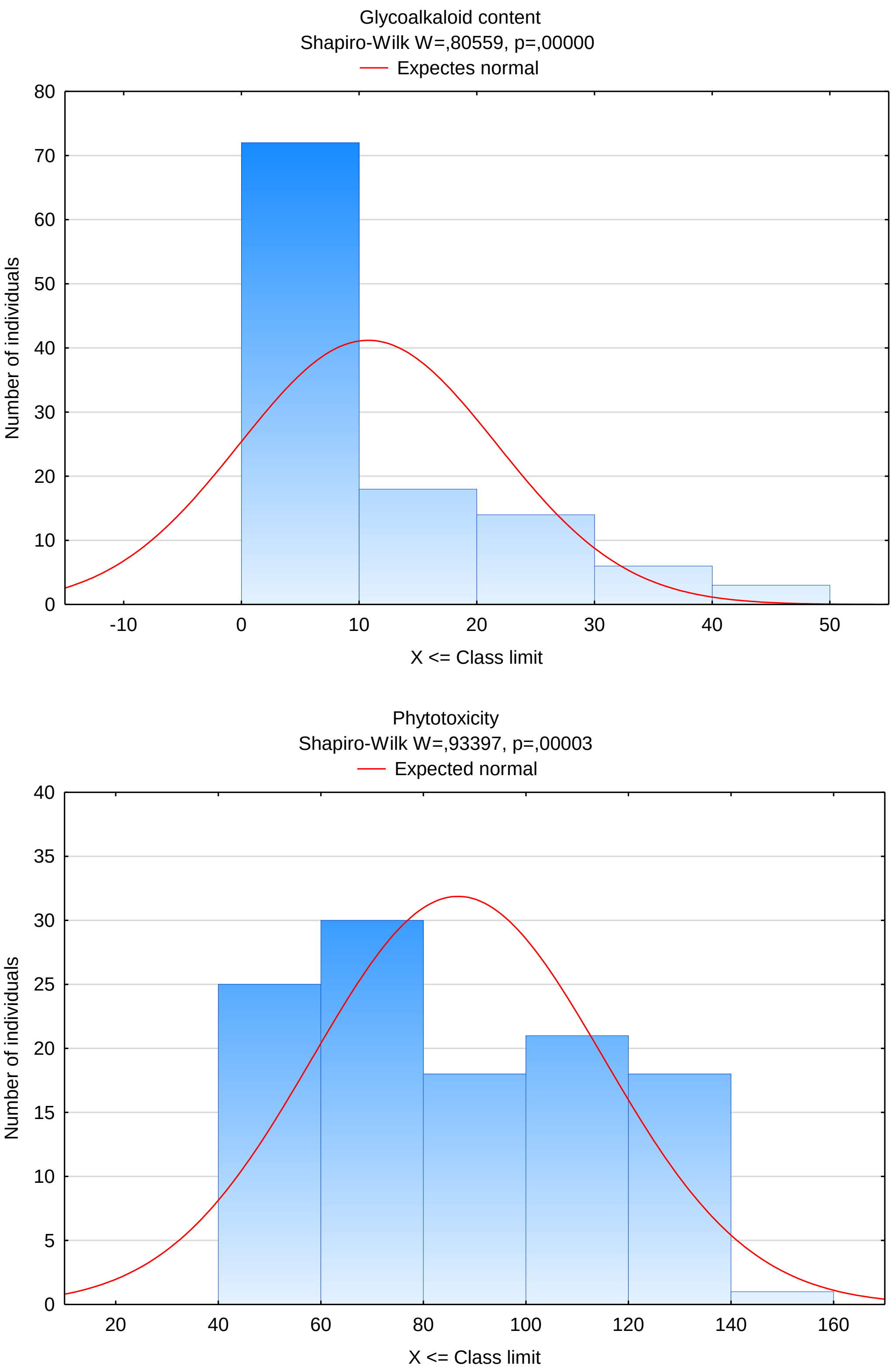

Supplementary Figure S1. Distribution of glycoalkaloid content and phytotoxicity in the 15-1 potato population.
