# Supplementary material for: Glycoalkaloid Composition and Flavonoid Content as Driving Forces of Phytotoxicity in Diploid Potato
Source: Int J Mol Sci. 2023 Jan 14;24(2):1657. doi: 10.3390/ijms24021657 (PMC9863746; doi:10.3390/ijms24021657)
Supplement: Supplementary file 1 [file ijms-24-01657-s001.zip › Supplementary Figure S2.pdf]

Supplementary Figure S2. Subgraphs of top 10 most enriched GO terms (p value ≤ 0.05) in BP category of DEGs in the (A) A' vs. B' and (B) A' vs. F' comparison groups.

A)

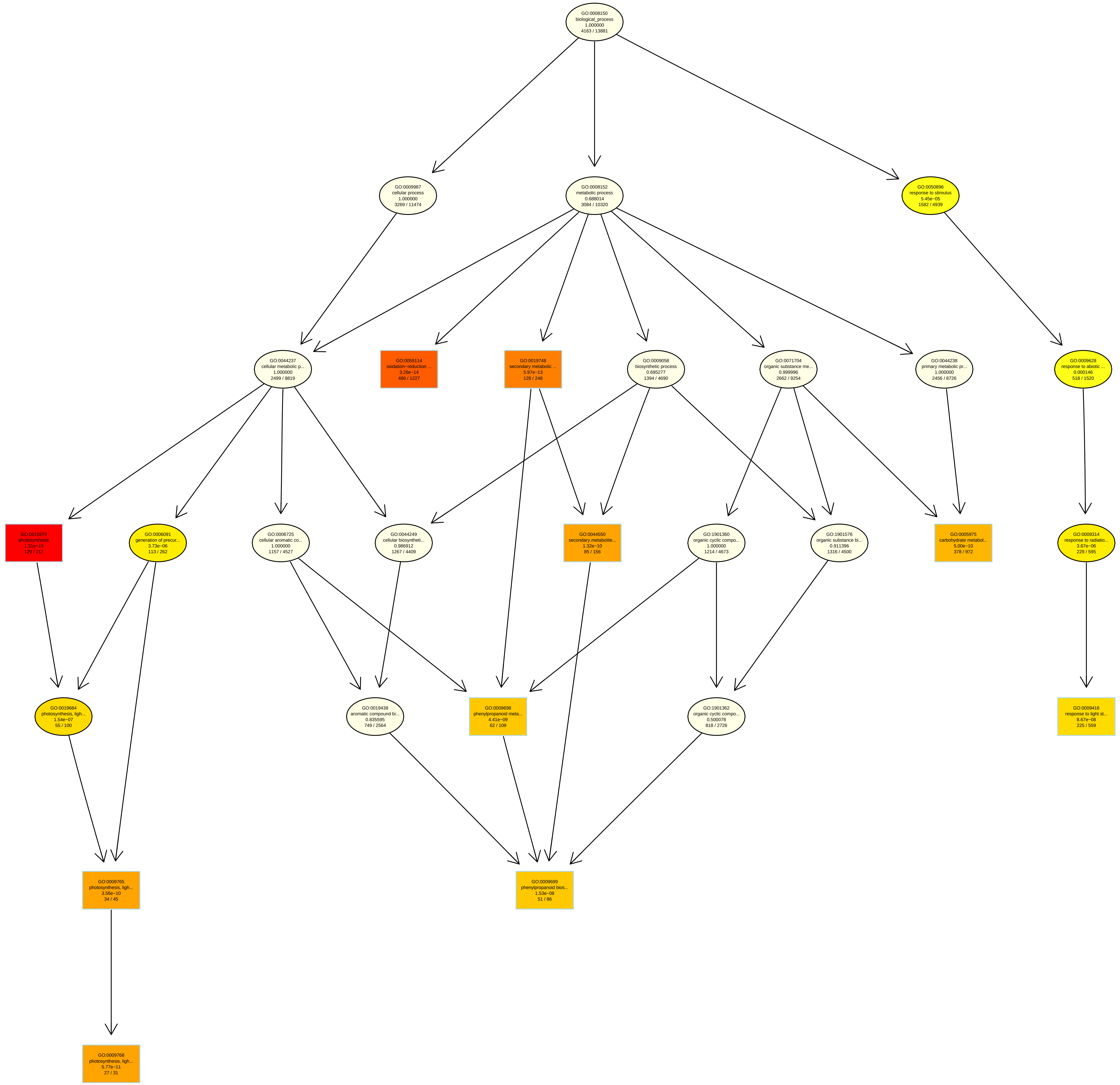

B)

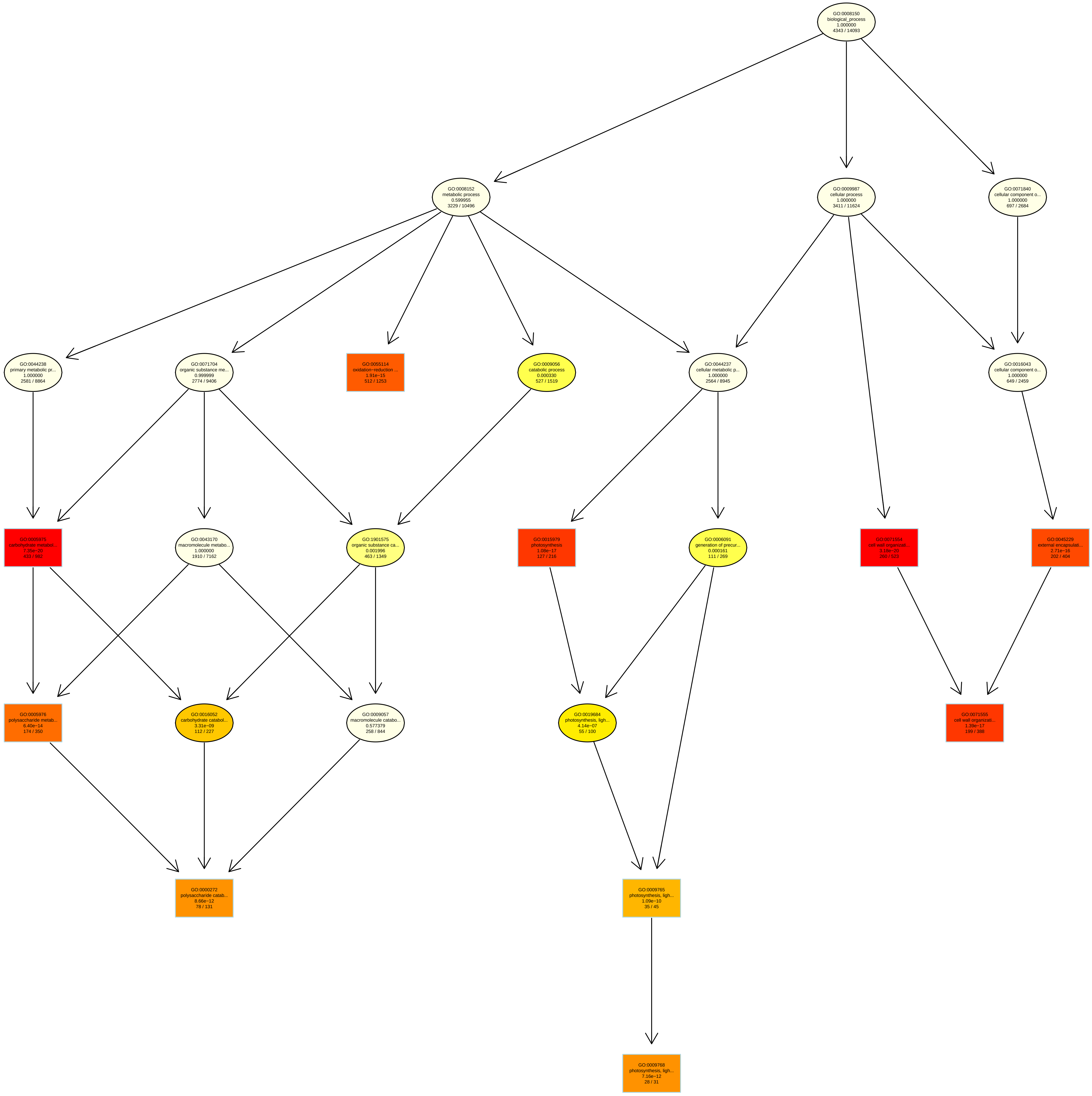
GO:0008150 biological_process 1.000000 4343 / 14093
GO:0008152 metabolic process 0.599955 3229 / 10496
GO:0009987 cellular process 1.000000 3411 / 11624
GO:0071840 cellular component o... 1.000000 697 / 2684
GO:0044238 primary metabolic pr... 1.000000 2581 / 8864
GO:0071704 organic substance me... 0.999999 2774 / 9406
GO:0055114 oxidation−reduction ... 1.91e−15 512 / 1253
GO:0009056 catabolic process 0.000330 527 / 1519
GO:0044237 cellular metabolic p... 1.000000 2564 / 8945
GO:0016043 cellular component o... 1.000000 649 / 2459
GO:0005975 carbohydrate metabol... 7.35e−20 433 / 982
GO:0043170 macromolecule metabo... 1.000000 1910 / 7162
GO:1901575 organic substance ca... 0.001996 463 / 1349
GO:0015979 photosynthesis 1.08e−17 127 / 216
GO:0006091 generation of precur... 0.000161 111 / 269
GO:0071554 cell wall organizati... 3.18e−20 260 / 523
GO:0045229 external encapsulati... 2.71e−16 202 / 404
GO:0005976 polysaccharide metab... 6.40e−14 174 / 350
GO:0016052 carbohydrate catabol... 3.31e−09 112 / 227
GO:0009057 macromolecule catabo... 0.577379 258 / 844
GO:0019684 photosynthesis, ligh... 4.14e−07 55 / 100
GO:0071555 cell wall organizati... 1.39e−17 199 / 388
GO:0000272 polysaccharide catab... 8.66e−12 78 / 131
GO:0009765 photosynthesis, ligh... 1.09e−10 35 / 45
GO:0009768 photosynthesis, ligh... 7.16e−12 28 / 31
